# First-in-Human Serum Stability Studies of [^177^Lu]Lu-AMTG: A Step Toward Improved GRPR-Targeted Radiopharmaceutical Therapy

**DOI:** 10.2967/jnumed.124.269132

**Published:** 2025-06

**Authors:** Veronika Felber, Nadine Holzleitner, Markus Joksch, Tim Suhrbier, Gunhild von Amsberg, Sarah Schwarzenböck, Jens Kurth, Martin Heuschkel, Thomas Günther, Bernd J. Krause

**Affiliations:** 1 Department of Chemistry, TUM School of Natural Sciences, Technical University of Munich, Garching, Germany;; 2 Department of Nuclear Medicine, Rostock University Medical Center, Rostock, Germany;; 3 Department of Oncology, University Medical Center Hamburg-Eppendorf, Hamburg, Germany; and; 4 Molecular Imaging Program at Stanford, Department of Radiology, School of Medicine, Stanford University, Stanford, California

**Keywords:** AMTG, in vivo serum stability, GRPR, first-in-human, ^177^Lu

## Abstract

The use of PET/CT with gastrin-releasing peptide receptor (GRPR) ligand [^68^Ga]Ga-AMTG has recently been shown to diagnose metastatic disease not detected by ^18^F-PSMA PET/CT in patients with metastatic castration-resistant prostate cancer. This study aimed to analyze the serum stability of [^177^Lu]Lu-AMTG in human subjects due to the compound’s high stability observed preclinically and to elucidate its therapeutic potential. **Methods:** Blood samples were collected at various time points after intravenous injection of 7.6 ± 0.1 GBq of [^177^Lu]Lu-AMTG and centrifuged. Serum samples were analyzed via reversed-phase high-performance liquid chromatography. **Results:** At 1 h after injection, the mean ± SD in vivo serum stability of [^177^Lu]Lu-AMTG was distinctly higher (62% ± 6%) than that of [^68^Ga]Ga-RM2 (19% ± 2%). **Conclusion:** Based on the high in vivo serum stability of [^177^Lu]Lu-AMTG in humans and favorable biodistribution, radiolabeled AMTG derivatives have the potential to improve radiopharmaceutical therapy for GRPR-expressing malignancies.

## Visual Abstract

**Figure:**
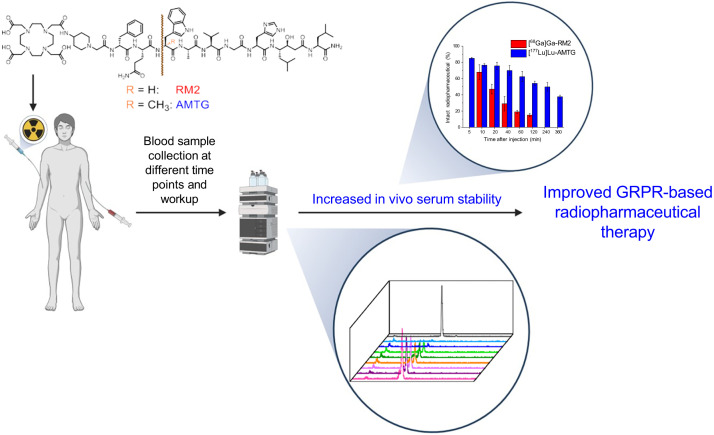

Based on the recent success of radiolabeled therapeutics, such as the prostate-specific membrane antigen (PSMA)–targeted compound [^177^Lu]Lu-PSMA-617 (Pluvicto; Novartis) (1,2), medical and industrial interest in nuclear medicine has increased substantially. Future research will focus on the development of radiopharmaceuticals that target biomarkers beyond PSMA (3). In addition to their high affinity, target uptake, favorable biodistribution profiles, and clearance kinetics, the in vivo serum stability of radiopharmaceuticals represents a key feature for successful treatment applications, as it affects bioavailability and, consequently, activity retention in target lesions (4).

Despite being investigated intensively in preclinical studies, in vivo serum stability of radiopharmaceuticals in humans is scarcely reported (5,6). Although small molecules (e.g., [^177^Lu]Lu-PSMA-617) and cyclic peptides (e.g., [^177^Lu]Lu-DOTATATE) were shown to be very stable (5,6), linear peptides (e.g., the gastrin-releasing peptide receptor [GRPR] ligands RM2 and NeoB) are typically more susceptible to enzymatic degradation. To date, no ^177^Lu-labeled GRPR ligand has been used in routine clinical practice, despite promising results observed in a first-in-human study of [^177^Lu]Lu-RM2 (7). This may be because only approximately 19% of [^68^Ga]Ga-RM2 was found to remain intact at 1 h after injection (8), potentially limiting the bioavailability of [^177^Lu]Lu-RM2 for therapeutic applications.

α-Me-L-Trp^8^-RM2 (AMTG) (Fig. 1) has displayed the highest preclinical in vivo stability among currently available radiolabeled GRPR ligands (9) and revealed favorable pharmacokinetics and GRPR-directed lesion uptake in a PET/CT scan of a patient with metastatic castration-resistant prostate cancer (mCRPC) who no longer showed sufficient PSMA expression (10). Multiple processes, such as epithelial-to-mesenchymal transition, are hypothesized to lead to a predominant neuroendocrine phenotype of prostate cancer, observed in approximately 17% of this patient cohort (11). Given the need for alternative treatment options for these patients, as well as for patients with other GRPR-expressing malignancies (e.g., breast cancer, glioblastoma multiforme), we evaluated the use of radiopharmaceutical therapy (RPT) with [^177^Lu]Lu-AMTG in 4 patients with mCRPC (12).

**FIGURE 1.**
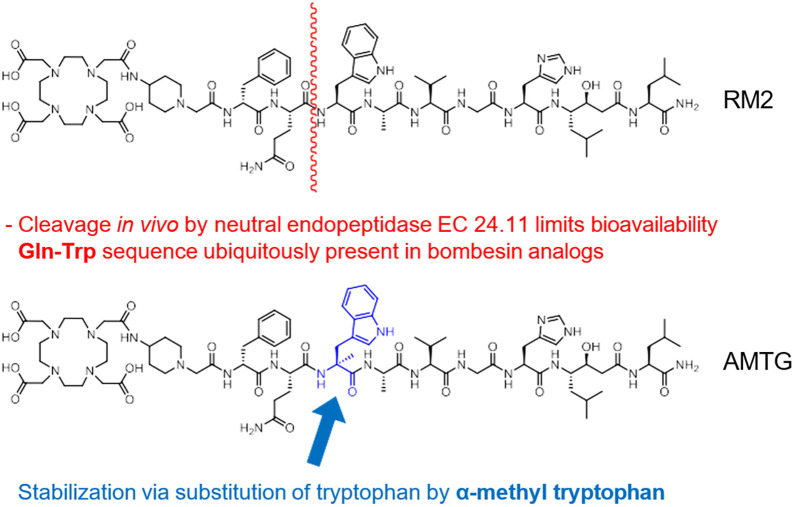
Chemical structures of RM2 and AMTG displaying major cleavage site (depicted in red) ubiquitously present in bombesin-based compounds and stabilizing moiety (α-methyl-L-tryptophan, depicted in blue).

Due to the significance of high in vivo serum stability for treatment applications, limited stability data available for radiopharmaceuticals, and promising preclinical in vitro and in vivo stability of [^177^Lu]Lu-AMTG, we investigated the in vivo stability and safety of [^177^Lu]Lu-AMTG RPT in 4 patients with mCRPC. We compared our results to the in vivo stability reported for [^68^Ga]Ga-RM2 (8), with the goal of developing an improved GRPR-based RPT.

## MATERIALS AND METHODS

All data are given as mean ± SD.

### Radiosynthesis

A detailed description of the synthesis of the precursor and the radiopharmaceutical is provided in the supplemental materials (available at http://jnm.snmjournals.org).

### RPT in Patients with mCRPC

[^177^Lu]Lu-AMTG RPT was administered as an individual medical treatment in 4 patients with mCRPC (mean age, 68 y; range, 52–79 y) who had exhausted all authorized treatment options. Patients who met the following criteria were considered for RPT: mCRPC or advanced prostate cancer with neuroendocrine differentiation; progressive disease after completion of standard treatment options (according to discretion of the treating urooncologist), including [^177^Lu]Lu-PSMA-617 RPT; and sufficient expression of GRPR in all known tumor lesions, as seen on [^68^Ga]Ga-AMTG PET/CT images. Due to the advanced, end-stage nature of the patients’ disease, exclusion criteria included renal insufficiency (estimated glomerular filtration rate < 30 mL/min/1.73 m^2^) and a severely impaired performance status (Eastern Cooperative Oncology Group score > 2). All patients provided informed consent to receive [^177^Lu]Lu-AMTG therapy with subsequent follow-up. Production and quality control of [^177^Lu]Lu-AMTG (investigational product) was performed according to good manufacturing practice regulations, and the therapies were used in accordance with the German Medicines Law, section 13(2b), and the Declaration of Helsinki, section 37.

The planned retrospective analyses of clinical data obtained during treatment were presented to the local ethics committee, who waived the need for a formal review (ethics committee at Rostock University Medical Center, file no. A 2024-0156). Analyses were performed anonymously in accordance with the Declaration of Helsinki and its later amendments.

### Preparation of Blood Samples and Stability Analysis

Blood samples (4–5 mL) were taken at 5, 10, 20, 40, 60, 120, 240, and 360 min after intravenous injection of 7.6 ± 0.1 GBq of [^177^Lu]Lu-AMTG and immediately centrifuged (6,000 rpm for 5 min). Plasma proteins of the decanted supernatant were precipitated by treatment with ice-cold acetonitrile (1:1 serum–acetonitrile mixture, v/v), followed by centrifugation (6,000 rpm for 5 min). Analysis of the ligand’s stability (contained in the decanted supernatant) was accomplished via reversed-phase high-performance liquid chromatography (RP-HPLC) (20%→35% acetonitrile in water [and 0.1% trifluoroacetic acid] in 20 min) equipped with a FlowStar^2^ LB 514 detector (Berthold Technologies GmbH & Co. KG).

## RESULTS

### Radiolabeling

^177^Lu-labeling was performed manually using 25 μg/GBq of AMTG precursor and 8.0–9.0 GBq of [^177^Lu]LuCl_3_, which resulted in radiochemical yields and purities exceeding 98% (Supplemental Fig. 1) and molar activities of 67 ± 3 GBq/μmol. All specifications were fulfilled (pH, 4–8; RP-HPLC retention times of [^177^Lu]Lu-AMTG and [^nat^Lu]Lu-AMTG matched [Supplemental Fig. 1], radiochemical purity ≥ 95.0%, content of unbound [^177^Lu]Lu-species < 3.0% [RP-HPLC] and < 3.0% [thin-layer chromatography]).

### In Vivo Serum Stability

**FIGURE 2.**
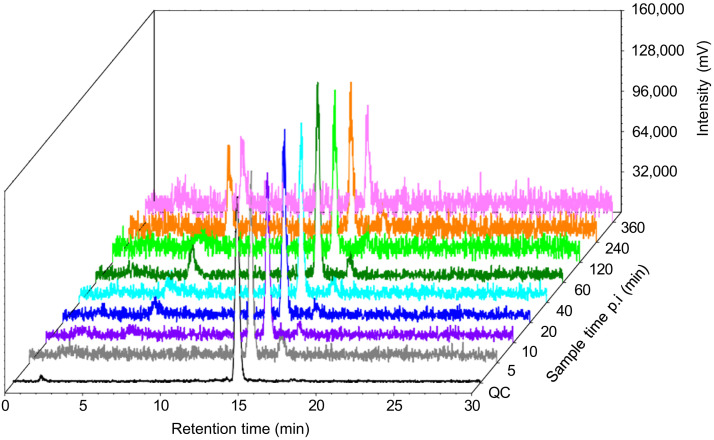
Exemplary radio–RP-HPLC chromatograms displaying intact [^177^Lu]Lu-AMTG (retention time, ∼14.5 min) as well as 2 metabolites (retention times, ∼5.4 and ∼15.7 min) in human serum over time. p.i. = postinjection; QC = quality control.

**FIGURE 3.**
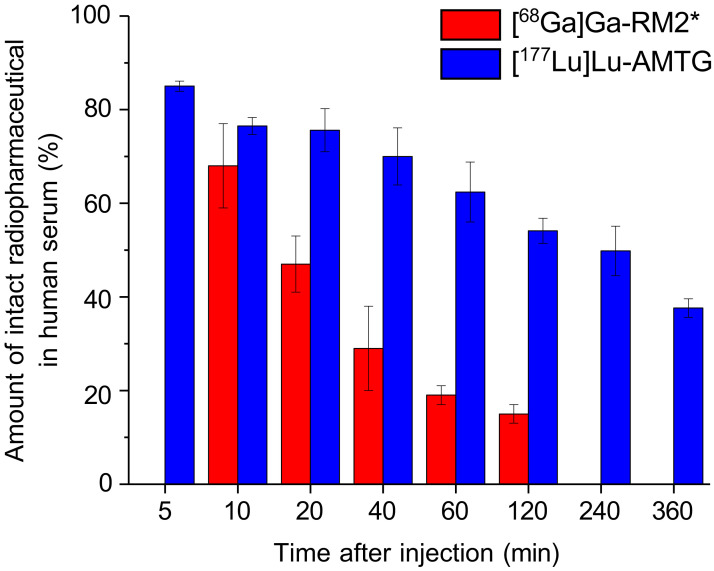
Comparison of in vivo stability of [^177^Lu]Lu-AMTG (*n* = 4; mean ± SD dose, 67 ± 3 GBq/μmol) and [^68^Ga]Ga-RM2 (*n* = 5; mean ± SD dose, ∼11 ± 3 GBq/μmol) in human serum at various time points after injection into human subjects. *Data from Roivainen et al. (8).

RP-HPLC chromatograms revealed a major peak for the intact [^177^Lu]Lu-AMTG, which highly corroborated that of the quality control run (before intravenous injection) and decreased over time (Fig. 2; Supplemental Fig. 2). Furthermore, 2 minor peaks were observed at each time point, with 1 peak increasing over time. Integration revealed that 85% ± 1% of the initially administered [^177^Lu]Lu-AMTG dose was still intact at 5 min after injection and decreased slowly over time (77% ± 2% at 10 min, 62% ± 6% at 60 min, and 38% ± 2% at 360 min) (Fig. 3; Supplemental Table 1). Nevertheless, in vivo stability of [^177^Lu]Lu-AMTG was higher than that of [^68^Ga]Ga-RM2 at each time point (68% ± 9% at 10 min, 19% ± 2% at 65 min, and 15% ± 2% at 150 min) (8).

## DISCUSSION

**FIGURE 4.**
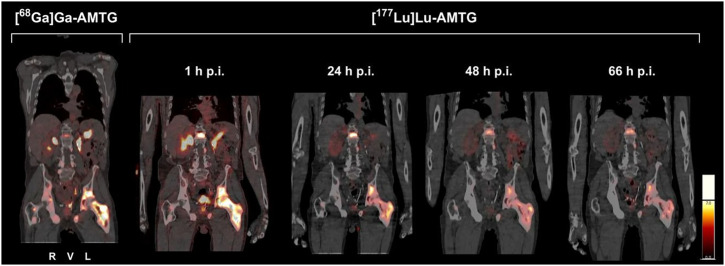
Uptake of [^68^Ga]Ga-AMTG and [^177^Lu]Lu-AMTG in 70-y-old patient with mCRPC with insufficient PSMA uptake and exhausted treatment options. Coronal section of pretherapeutic [^68^Ga]Ga-AMTG PET/CT image (left) displays pronounced uptake in osseous metastases, including in ilium and femoral neck and shaft. Intratherapeutic [^177^Lu]Lu-AMTG SPECT/CT image (right) at 1, 24, 48, and 66 h after injection shows intense and persistent activity retention in these lesions. All images were identically scaled to SUVs. p.i. = postinjection.

Due to the therapeutic potential and suggested lack of sufficient in vivo stability of currently used radiolabeled GRPR ligands, we developed AMTG, a peptide chemically stabilized to reduce enzymatic degradation. [^68^Ga]Ga-AMTG PET/CT has previously demonstrated a high tumor-to-background ratio, with relevant tracer accumulation observed only in the pancreas and minimal uptake noted in other organs at risk, such as the kidneys and salivary glands (10). Although these organs are commonly affected by other RPTs targeting PSMA or the somatostatin-2 receptor, among others, [^68^Ga]Ga-AMTG and [^177^Lu]Lu-AMTG displayed lower rates of accumulation in these organs (Fig. 4). Moreover, rapid activity clearance from the pancreas was observed (12), which highlights the therapeutic potential of [^177^Lu]Lu-AMTG. We examined the clinical value of [^177^Lu]Lu-AMTG versus GRPR-based RPT by analyzing the in vivo serum stability of [^177^Lu]Lu-AMTG in the study cohort.

Synthesis of [^177^Lu]Lu-AMTG according to good manufacturing practice was achieved within 25 min and resulted in similar radiochemical yields and molar activities to those reported for [^177^Lu]Lu-RM2 (7). No adverse effects or changes in blood pressure, body temperature, heart rate, or general well-being were observed after administration of [^177^Lu]Lu-AMTG, during or after blood sample collection, or during the following days of treatment.

Although [^177^Lu]Lu-AMTG was infused over 30 min (flow rate, 0.6 mL/min), we defined the starting point of this study as 15 min after the start of the injection. For example, stability measured 5 min after injection would include a mixture of [^177^Lu]Lu-AMTG that had already been metabolized for up to 20 min and a small fraction of [^177^Lu]Lu-AMTG that had been metabolized for less than 5 min. Compared with a bolus injection, administration via infusion led to an apparent lower stability at early time points. However, the influence on later time points appeared to be less pronounced. No signals of intact compound or metabolites could be detected in blood samples taken later than 6 h after injection by the radioactivity detector, likely attributable to the fast clearance of [^177^Lu]Lu-AMTG.

The blood sample work-up was fast and simple. Despite initial treatment with ice-cold acetonitrile and centrifugation (6,000 rpm for 5 min), some supernatants required further treatment with acetonitrile (1:1 supernatant–acetonitrile mixture, v/v) and centrifugation (13,000 rpm for 5 min) before radio–RP-HPLC analysis. RP-HPLC chromatograms displayed 3 peaks over time, which corroborated our preclinical data (9). One peak (retention time, ∼15 min) was attributed to intact [^177^Lu]Lu-AMTG and another peak (retention time, ∼5 min) was attributed to the major metabolite. The third peak (retention time, ∼16 min), present at each time point and showing a similar interval over time, might represent an intermediate compound that is initially generated from [^177^Lu]Lu-AMTG and further metabolized to the major metabolite.

In accordance with our preclinical data (9), distinctly higher in vivo serum stability over time was observed for [^177^Lu]Lu-AMTG compared with [^68^Ga]Ga-RM2 (3.3-fold higher percentage at 1 h after injection) (8). A comparison of serum stability of [^177^Lu]Lu-AMTG with [^177^Lu]Lu-RM2 would be more appropriate; however, to the best of our knowledge, no data on serum stability of [^177^Lu]Lu-RM2 or other GRPR-targeted compounds in humans have been reported. Moreover, the noticeably lower molar activity applied for [^68^Ga]Ga-RM2 (∼11 ± 3 GBq/μmol) (8) compared with [^177^Lu]Lu-AMTG (67 ± 3 GBq/μmol in this study) would likely lead to lower in vivo serum stability of [^177^Lu]Lu-RM2 if administered with similar molar activity to [^177^Lu]Lu-AMTG, as a higher number of unlabeled peptide (being metabolized as well) hampers the radiopharmaceutical’s enzymatic degradation. Although the in vivo serum stability of [^177^Lu]Lu-AMTG (linear peptide) is lower than that reported for [^177^Lu]Lu-PSMA-617 (small molecule) (6) or [^177^Lu]Lu-DOTATATE (cyclic peptide) (5), a substantial improvement over currently used GRPR ligands was observed, indicating improved GRPR-based RPT.

This study had several limitations. One limitation was the comparison of [^177^Lu]Lu-AMTG with [^68^Ga]Ga-RM2, as differing molar activities and injection velocities were applied. Furthermore, stability was determined only up to 6 h after injection of [^177^Lu]Lu-AMTG, as the radioactivity detector was not sensitive enough to detect the low amount of radioactivity present in blood at 24 h. This could be resolved by the collection of fractions during the RP-HPLC run and by quantification via γ-counter in future studies. Another limitation was the small size of the cohort (4 patients). However, as we compared our study to a study evaluating the in vivo stability of the structurally similar GRPR ligand RM2 in 5 healthy human subjects (8), we believe our cohort size was sufficient for comparative purposes. Future studies should evaluate [^177^Lu]Lu-AMTG dosimetry, toxicity, and biodistribution data.

## CONCLUSION

[^177^Lu]Lu-AMTG RPT was shown to be safe in 4 patients with mCRPC, and its improved in vivo serum stability, when compared with currently used GRPR ligands, might lead to more-effective GRPR-based RPT. This could be particularly relevant for the treatment of mCRPCs that lack sufficient PSMA expression, as well as breast cancer and glioblastoma multiforme. We anticipate that future studies for novel radiopharmaceuticals will also focus on determination of metabolic stability in human subjects, given the robust and fast experimental procedure described herein.

## DISCLOSURE

A patent application on modified GRPR-targeted ligands, including AMTG, with Thomas Günther as coinventor, has been filed. Bernd J. Krause serves as an advisor (Terumo, Rotop, AAA/Novartis, PSI CRO, ITM, Bayer, and Janssen) and receives third-party funding (AAA/Novartis, AMGEN, and Eisai), travel support (AAA/Novartis), and royalties (AAA/Novartis, Bayer, and Janssen). Jens Kurth received remuneration as a member of advisory boards (Novartis and GE HealthCare). No other potential conflict of interest relevant to this article was reported.

## Notes

KEY POINTS **QUESTION:** Is the high in vivo serum stability observed preclinically for [^177^Lu]Lu-AMTG reflected in the clinical setting? **PERTINENT FINDINGS:** [^177^Lu]Lu-AMTG revealed high in vivo serum stability over time in 4 patients with mCRPC, which, together with its high retention in lesions and rapid clearance kinetics from the pancreas, makes it a promising tool for GRPR-based RPT. **IMPLICATIONS FOR PATIENT CARE:** Although dosimetry data remain to be determined, particularly with regard to a potential dose-limiting toxicity to the pancreas, [^177^Lu]Lu-AMTG is expected to improve RPT in patients with GRPR-expressing malignancies because of its high in vivo serum stability and bioavailability.
